# Rapid magnetic separation: An immunoassay platform for the SERS-based detection of subarachnoid hemorrhage biomarkers

**DOI:** 10.3389/fchem.2022.1002351

**Published:** 2022-10-21

**Authors:** Ying Wang, Jingyi Sun, Peng Zhao, Hui Yi, Hui Yuan, Mingfeng Yang, Baoliang Sun, Fengyuan Che

**Affiliations:** ^1^ Linyi People’s Hospital, Shandong First Medical University & Shandong Academy of Medical Sciences, Taian, Shandong, China; ^2^ Shandong Provincial Hospital, Shandong First Medical University & Shandong Academy of Medical Sciences, Jinan, Shandong, China; ^3^ Second Affiliated Hospital, Shandong First Medical University & Shandong Academy of Medical Sciences, Taian, Shandong, China

**Keywords:** subarachnoid hemorrhage, blood-brain barrier, double detection, surface enhance Raman scattering, biosensors

## Abstract

The blood–brain barrier (BBB) is of vital importance to the progression and prognosis of subarachnoid hemorrhage (SAH). The construction of a simple, sensitive, and accurate detection assay for measuring the biomarkers associated with BBB injury is still an urgent need owing to the complex pathogenesis of SAH and low expression levels of pathological molecules. Herein, we introduced surface-enhanced Raman scattering (SERS) label-embedded Fe_3_O_4_@Au core-shell nanoparticles as ideal SERS sensors for quantitative double detection of MMP-9 and occludin in SAH patients. Meanwhile, utilizing the SERS signals to dynamically estimate MMP-9 and occludin concentration in the rat SAH model is the first application in exploring the relationship of pathological MMP-9 and occludin molecular levels with neurobehavioral score. This method warrants reliable detection toward MMP-9 and occludin with a wide recognition range and a low detection limit in blood samples. Furthermore, the results monitored by the SERS assay exactly matched with those obtained through a traditional enzyme-linked immunosorbent assay (ELISA). The aforementioned results demonstrated this novel biosensor strategy has extensive application prospects in the quantitative measurement of multiple types of biomolecules in body fluid samples.

## Introduction

Subarachnoid hemorrhage (SAH) is a relatively rare and critical subtype of stroke, threatening patients even under 55 years of age, placing a heavy burden on individuals and society due to its high disability and fatality rate (Sehba et al., 2012; Macdonald and Schweizer 2017). Patients with SAH usually have a combination of symptoms (Al-Shahi et al., 2006). The delayed diagnosis of SAH in some cases with atypical presentations may endanger the life of these patients to some degree; a false-negative computed tomography (CT) brain scanning within the first hours after SAH represents another problem; on the other hand, early treatment can improve the outcome of patients; for these reasons, the importance of timely, fast, and simple diagnosis of SAH is stressed (Andrioli and Cavazzani 1998; de Gans et al., 2002; Benninger et al., 2015). Studies have shown that early brain injury and immune response occur after SAH, prompting many biochemical molecules and inflammatory factors to be released into the cerebrospinal fluid (CSF) and peripheral blood by damaging the blood–brain barrier (BBB) (van Gijn and Rinkel 2001; Atsev and Tomov 2020). The increase of these substances can be used as biomarkers of SAH, which is conducive to early diagnosis and treatment of SAH, thereby improving the patients’ prognosis.

Matrix metalloproteinase-9 (MMP-9) belongs to the matrix metalloproteinase (MMP) family of zinc-containing endopeptidases and was identified to be involved in the pathophysiology of various neurological diseases, including SAH (Vafadari et al., 2016). Activation of MMP-9, which is usually secreted as zymogen proform by neurons, endothelial, and glial cells, can lead to BBB disruption after SAH because of degradation of tight junction proteins responsible for BBB integrity, contributing to cerebral edema and neuronal apoptosis (Horstmann et al., 2006; Guo et al., 2015). Previous research studies revealed that elevated levels of MMP-9 in the blood and CSF were associated with SAH-induced vasospasm and a poor clinical outcome at 3 months (McGirt et al., 2002; Chou et al., 2011).

Occludin, as one of main components of the tight junction transmembrane proteins, together with claudin-5 and zona occludens-1 (ZO-1), plays a critical role in ensuring the function of BBB (Altay et al., 2012; Keep et al., 2018; Liu et al., 2020). Recent studies indicated that damage of BBB integrity and increase of paracellular permeability can be found after SAH, which is caused by MMP-9-induced degradation of occludin with some inflammatory cytokines and pathologic molecules entering into the brain parenchyma (Chen et al., 2014; Ying et al., 2016; Shi et al., 2017).

Aiming at the aforementioned two biomarkers closely related to BBB damage after SAH, we proposed to use magnetic separation combined with surface-enhanced Raman scattering (SERS) to detect their content in blood. SERS is a powerful measuring technique for detecting trace amounts of multiple analytes (including nucleic acids, proteins, biomarkers, and small molecules) with high sensitivity, specificity, and spectral resolution (Macdonald and Schweizer 2017). For SERS, amplification of the Raman signal can reach 10 to 14 orders of magnitude and generate electromagnetic field enhancement spots that are called “hot spots”, which evolve from the gaps of two neighboring metal nanoparticles or the rough surface of metal nanoparticles (Lee et al., 2015; Ding et al., 2016; Lee et al., 2019). Compared with other detection techniques, SERS has many obvious advantages, including narrow spectral bandwidth, unique fingerprint spectra, low photobleaching, and little confounding background signals (Zhai et al., 2012; Ma et al., 2017; Ye et al., 2017).

In order to gain SERS signals from Raman active elements, multiple nanomaterials have been prepared. Studies show that the gaps between metal core-shell structures can produce a stable and intense electromagnetic field (Khlebtsov et al., 2020). Gold-modified magnetic nanoparticles (AuMNPs) are the nanomaterials that display great potential for detection analysis, which is not only due to the special magnetic property and biocompatibility of the magnetic nanoparticles but also attributed to feasibly biochemically modified and conjugated gold nanoparticles outside the AuMNPs (Wang et al., 2016; Wang et al., 2018). In addition, the magnetic core facilitates the materials separated from the fluid by an external magnet (Yang et al., 2021). Meanwhile, we fabricated an SERS sensor based on antibody-functionalized gold nanostars (GNSs) for sensitive detection of the levels of MMP-9 and occludin in the blood after SAH.

**SCHEME 1:**
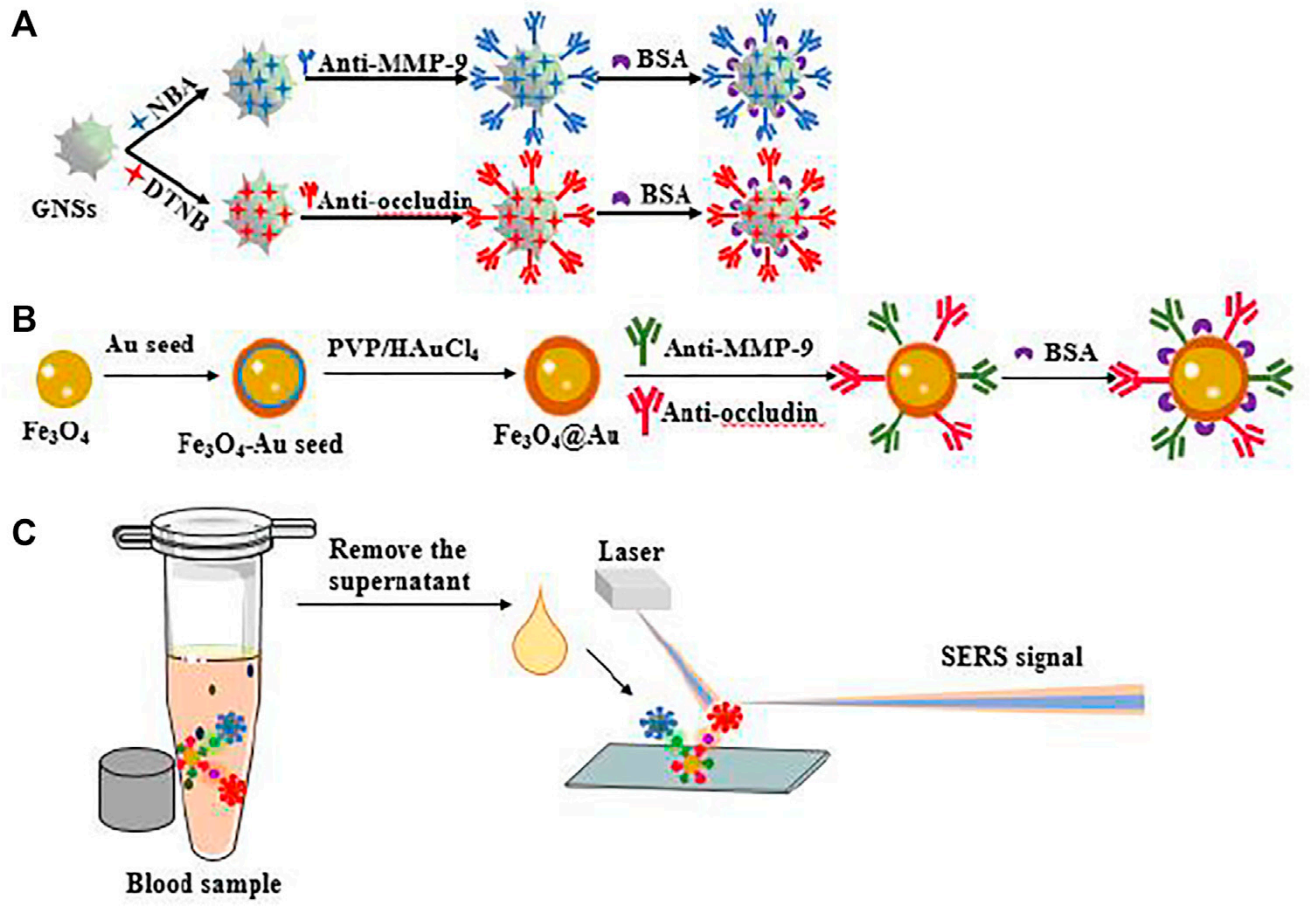
**(A)** Schematic illustration of the synthesis of GNS tags and **(B)** AuMNPs@MMP-9@occludin. **(C)** Application for SERS sensor detection of MMP-9 and occludin in blood samples.

Herein, antibody-functionalized GNS tag SERS sensor combined with magnetic separation provided for ultrasensitive double detection of MMP-9 and occludin in the blood. The anti-MMP-9 and anti-occludin antibody were simultaneously attached to AuMNPs and were paired by carboxylation of magnetic beads. Then, the GNS tags could exactly combine with the antibody on the surface of AuMNPs, and the combined complex is submitted to the SERS sensor for detection, so the application of magnetic bead characteristics can further save the amount of nanomaterials and the process of modified glass substrates with materials (Scheme 1). This sensor method is simple, fast, and sensitive for the detection of blood components. Thereby, it can play a powerful predictive role in the evaluation of disease severity and prognosis, thereby improving the clinical outcome of SAH patients.

## Experimental section

### Materials and equipment

Trisodium citrate, chloroauric acid (HAuCl_4_), hydroquinone, ethanol, ethylene glycol, and bovine serum albumin (BSA) were acquired from Shanghai Chemical Reagent Co., Ltd., China. Ferric chloride hexahydrate (FeCl_3_·6H_2_O), sodium acetate trihydrate, phosphate-buffered saline (PBS), and 5, 5′-dithiobis-(2-nitrobenzoic acid) (DTNB) were purchased from Solarbio Co., Ltd. (Beijing, China). 3-Aminopropyl triethoxysilane (APTES), N-hydroxysuccinimide (NHS), 1-ethyl-3-(3-(dimethylamino)-propyl) carbodiimide (EDC), dimercaptosuccinic acid (DMSA), and Nile Blue A (NBA) were purchased from Aladdin Ltd. (Shanghai, China). MMP-9, occludin, monoclonal occludin antibody, mouse IgG, and anti-human MMP-9 were purchased from Abcam. The MMP-9 enzyme-linked immunosorbent assay (ELISA) kit and occludin ELISA kit were purchased from Abcam.

The ultraviolet–visible–near infrared (UV–Vis–NIR) spectroscopy images were captured using a Shimadzu UV-3600 plus spectrophotometer. Scanning electron microscopy (SEM) images were acquired via using a scanning electron microscope (FE-SEM, GeminiSEM 300, Carl Zeiss, Germany). The transmission electron microscopy (TEM) analysis images were obtained by utilizing a JEM 1200EX transmission electron microscope (JEOL, Japan). SERS spectrum analysis was carried out using a Horiba scientific Raman spectrometer (Xplora Plus) applying a ×100 objective lens and a 785-nm He–Ne excitation laser source.

### Preparation of blood samples from the rat SAH model and SAH patients

Male Sprague–Dawley (SD) rats (280–300 g, 10 weeks) were provided by Pengyue Laboratory Animal Breeding Co., LTD., China, and all procedures were performed under the guidelines of the Use of Experimental Animals of National Institutes of Health (Zhu et al., 2018). The rat SAH model was constructed according to the intracranial endovascular perforation method (Zhang et al., 2018). The SAH grading system was used for assessing the experimental SAH, and rats with mild grades (score of ≤8 points) were excluded from the trial (Sugawara et al., 2008). Its scoring was given depending on the amount of blood in the basal cistern, which was segmented into six regions that were graded as follows: 0 means no subarachnoid hemorrhage, 1 means minimal subarachnoid hemorrhage, 2 means moderate subarachnoid hemorrhage, and 3 means all arteries were covered with blood clots within the regions.

The rat blood samples were gained through cardiac puncture and then centrifuged at 2,000 rpm for 10 min. The obtained supernatant was clustered for ELISA assay. However, for the SERS analysis, we used the whole blood samples. In addition, the blood samples of SAH patients were supported by the Second Affiliated Hospital of Shandong First Medical University. We collected blood from patients at 6, 12, 24, 48, and 72 h following SAH. Human blood samples were also processed as per the aforementioned centrifugation and then detected via ELISA and SERS analyses. All procedures were approved by the Ethics Committee of the Second Affiliated Hospital, Shandong First Medical University.

### Neurobehavioral assessment

The neurobehavioral assessment of a rat was performed blindly involving the modified Garcia scoring system and Morris water maze (MWM) test. In order to assess the neurological function score, the previously reported modified Garcia scoring system was used, which included six tests with a total score of 18 (Sugawara et al., 2008). In this experiment, we evaluated the rat SAH model after 24 h. We also carried out the MWM test to estimate the spatial memory at 7 days after SAH, which contained two parts: 5-day spatial acquisition and 1-day probe trial (Sugawara et al., 2008; Xie et al., 2015; Xie et al., 2018). For the aforementioned neurobehavioral trials, 24 rats were randomly divided into the sham group and SAH group, with six rats for each trial group.

### Synthesis of urchin-like gold nanostars and AuMNPs

In this experiment, urchin-like gold nanostars (GNSs) were synthesized by a seed-mediated method (Xia et al., 2017). Specifically, 300 μl of HAuCl_4_ (1%) and 900 μl of trisodium citrate (1%) were added into the prepared 30 ml of boiling water. The reaction solution was heated continuously until the color changed from transparent to red wine. Then, the seed solution can be used when it cooled to room temperature. To synthesize the GNSs, 100 μl of HAuCl_4_ (1%) was mixed with 9.6 ml H_2_O into a glass vial at room temperature with gentle stirring (650 rpm). Subsequently, 50 μl above the prepared seed solution was added in the mixture. After the solution was stirred evenly, 22 μl trisodium citrate (1%) and 500 μl hydroquinone (30 mM) were injected in the reaction system with stirring. In order for the reaction to proceed completely, stirring was continued for another 30 min. Finally, the resulting GNSs were stored at room temperature in the form of a storage solution.

The preparation of Fe_3_O_4_@Au (AuMNPs) was also conducted through the seed-mediated method by using Fe_3_O_4_ nanoparticles, which were composed by using the solvothermal reaction. Briefly, to ensure that the PEI integrates with Fe_3_O_4_, the mixture of 10 ml 1 mg/ml pofyethyleneimine (PEI) aqueous solution and 0.1 g of Fe_3_O_4_ was sonicated for 15 min. Then, another 30 min of sonication was performed after adding the aforementioned prepared Fe_3_O_4_ into 50 ml of Au nanoparticles, and subsequently, the Fe_3_O_4_@Au seed was generated. After that, the seed was dispersed into a 100-ml mixture aqueous solution of NH_2_OHHCl (0.5 mg/ml) and polyvinyl pyrrolidone (PVP) powder (300 mg) and then sonicated for 15 min. The aforementioned solution was mixed with 40 μM of HAuCl_4_·4H_2_O. After another 15 min of sonication, the final products were washed with ultrapure water twice and re-dispersed into ethanol for further use.

### Preparation of MMP-9 and occludin labeling antibody-modified SERS active GNS tags

The antibody-modified GNS tags consist of the MMP-9 labeling antibody-modified GNSs (GNSs@DTNB@MMP-9) and GNSs@NBA@occludin. The preparation of the SERS tags was as follows: First, 1 ml GNSs dispersed in 10 ml of double-distilled water were prepared for two portions. Then, 300 μl of 10^−2^ M DTNB aqueous solution and 300 μl of 10^−2^ M NBA aqueous solution were incubated in the aforementioned two beakers, respectively, at room temperature for 1–2 h. Subsequently, the mixture of 50 μl EDC (1 mM) and 50 μl NHS (1 mM) was added into the reaction system for activating the carboxyl groups of GNSs about 1 h. After that, the GNSs@DTNB were co-incubated with 10 μl of MMP-9 labeling antibody (10 ng/ml), and the GNSs@NBA were co-incubated with the occludin-labeling antibody (10 ng/ml) for 1–2 h. Finally, the aforementioned two solutions were further processed with 100 μl 1% BSA solution for 1 h to block the positive sites of the antibody.

### Preparation of MMP-9 and occludin coating antibody-modified SERS label-embedded AuMNPs

For modifying the AuMNPs with MMP-9 and the occludin-coating antibody, we applied the method of peptide bond formation between them. During preparation, 10 μl AuMNPs were ultrasonically mixed into 1 ml of the APTES solution for 30 min to modify anmino groups on the surface of AuMNPs. Thereafter, AuMNPs were separated from the aforementioned mixture by magnetic separation and then re-dispersed into 2 mM DMSA solution for 4 h. After the mixed solution was washed with PBS once and re-dissolved in 1 ml of the PBS solution, the mixture of 50 μl EDC (1 mM) and 50 μl NHS (1 mM) was added into the aforementioned solution for activating the carboxyl groups of AuMNPs for about 1 h. The obtained product was washed once again with PBS and then re-dispersed in 1 ml of the PBS solution. Subsequently, MMP-9 and the occludin-coating antibody were co-incubated into the reaction system for 1–2 h. At last, 100 μl 1% BSA blocking was added into the aforementioned system to cover the positive sites of the antibody.

### SERS sensors for double detection of MMP-9 and occludin

Before SERS sensor detection of MMP-9 and occludin, we tested the SERS signal of GNSs@NBA and GNSs@DTNB for evaluating the property of GNSs. Furthermore, the SERS signal of AuMNPs@MMP-9, AuMNPs@occludin, and AuMNPs@MMP-9@occludin was also examined for characterizing their property. The spectra were gained from ten different sites of each sample, and the average was applied as the final SERS result.

The process for double detection of MMP-9 and occludin through a Raman spectrometer was conducted as follows: 20 μl GNSs@NBA SERS tags, 20 μl GNSs@DTNB SERS tags, 20 μl AuMNPs @MMP-9@occludin, MMP-9, and occludin were mixed in 100 μl of PBS solution. The reaction solution was incubated at room temperature for 2 h to guarantee an adequate response between the tags and the antibody-modified SERS label-embedded AuMNPs. Then, the purification of GNS–AuMNP assemblies was fulfilled via magnetic separation. Thereafter, the obtained precipitates were re-suspended in 100 μl double distilled water. In this study, the SERS spectra were measured through a Raman microscope equipped with a 785-nm laser and an accumulation time of 2 s. For detecting the levels of MMP-9 and occludin, we adopted the standard curve of correlation between SERS intensity and the logarithm of the target concentration by adding different concentrations of MMP-9 and occludin. The outcome of the SERS spectra from the testing blood samples was compared with that of the aforementioned reference standard curve and generated the concentration value of MMP-9 and occludin.

## Results and discussion

### Characterization of GNSs

To inspect the morphology and structure of the GNSs, the SEM technique was usually adopted. Supplementary Figure S1A shows the SEM analysis image of GNSs, revealing that numerous nanostructures with dendrites were gained, which possessed uniform shapes and particle sizes. The specific size distribution was further analyzed by using dynamic light scattering (DLS) measurement, which indicated that most of its particle size was around 100 nm, and no significant changes were found before and after modifying the signal molecule. (Supplementary Figures S1B,C). The optical characteristics of GNSs were explored by the UV–Vis–NIR absorption spectrometer system with the results presented in Supplementary Figure S1D. The recording indicated that the UV–Vis–NIR absorption spectra of GNSs, NBA-labeled GNSs, and DTNB-labeled GNSs had similar sharp and strong LSPR peaks at around 700 nm. For SRES signal detection, we selected NBA and DTNB as the Raman indicators in this study and the characteristic SERS spectrum bands of NBA-labeled GNSs and DTNB-labeled GNSs located at nearly 592 cm^−1^ and 1,332 cm^−1^ respectively, whose results are shown in Supplementary Figures S1E, F. The detailed calculation about the SERS enhancement factor of nanoprobes is presented in Supplementary Figure S2.

### Characterization of AuMNPs

**FIGURE 1:**
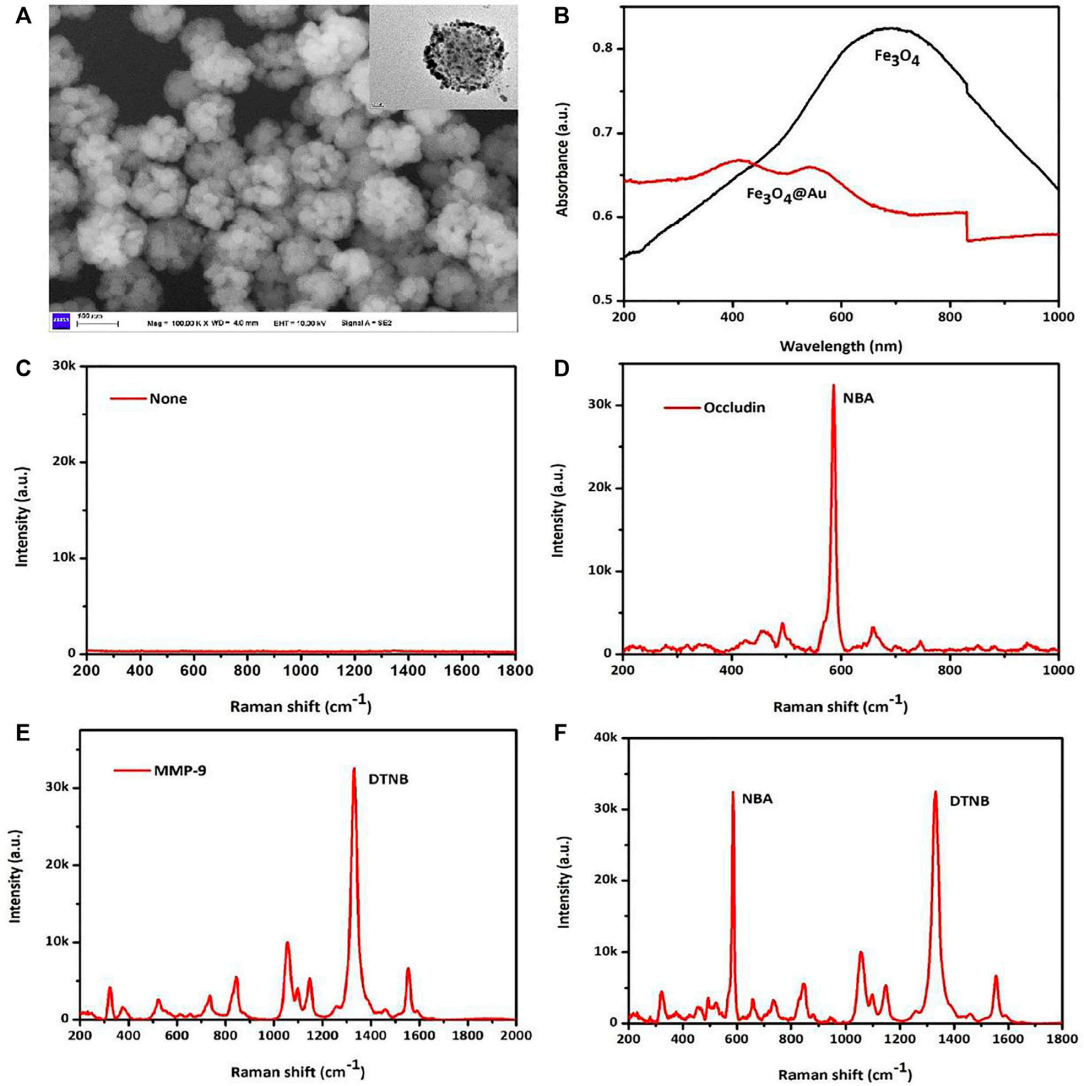
**(A)** SEM images and TEM images of AuMNPs. **(B)** UV–Vis–NIR spectra of Fe_3_O_4_@Au and pure Fe_3_O_4_. **(C)** SERS spectra of not added MMP-9 and occludin. **(D)** SERS spectra of only added occludin. **(E)** SERS spectra of only added MMP-9. **(F)** SERS spectra of added occludin and MMP-9.

SEM and TEM techniques were applied to illustrate the structural characteristics of AuMNPs, which is presented in Figure 1A. The SEM of AuMNPs showed that they were dispersed in a colloidal solution with a uniform size of 100–150 nm, with the TEM in the upper right corner depicting the two layers of the Fe_3_O_4_ magnetic core with a gold shell. The optical characteristics of AuMNPs and pure Fe_3_O_4_ that are described in Figure 1B revealed that the UV–Vis–NIR absorption spectrum of AuMNPs was relatively wide and weak LSPR band at 400 nm, while the strong absorption peak of pure Fe_3_O_4_ was located at around 700 nm. Here, to discuss the feasibility of the experiment, when occludin and MMP-9 did not exist, the SERS sensor detected no signal; when only MMP-9 exists, only the DTNB signal and signal stability is found; when there is only occludin, only the NBA signal and signal stability is found; when both exist, the SERS sensor detects the two signals, and the two signals are interfering, easy to distinguish, and show signal stability (Figures 1C–F).

### SERS assay for MMP-9 and occludin concentrations in PBS solution

**FIGURE 2:**
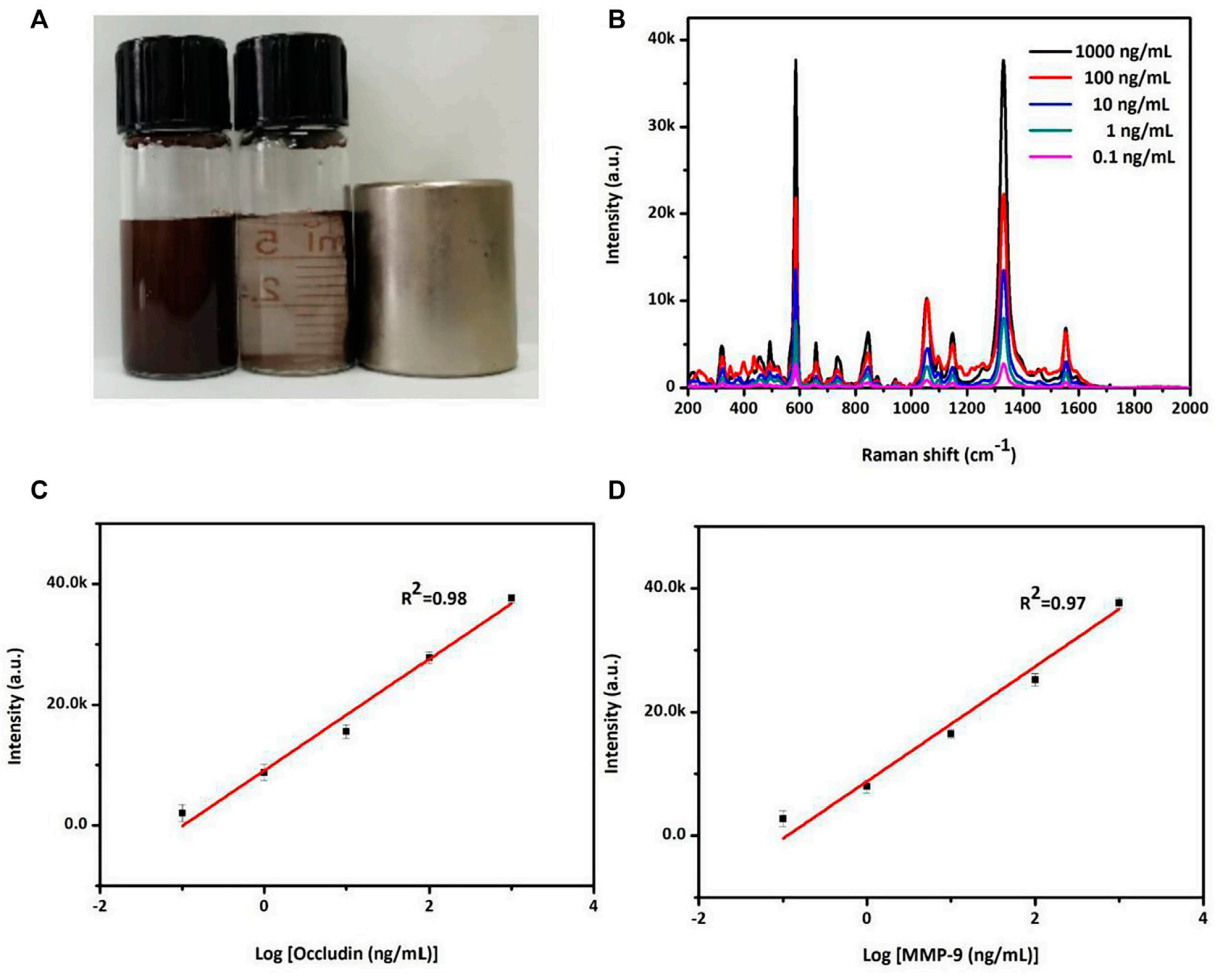
**(A)** Photograph of magnetic separation for the MNPs. **(B)** SERS spectra of NBA corresponding to the various concentrations of occludin and DTNB corresponding to the various concentrations of MMP-9 in a PBS solution. **(C)** Standard curve of the relationship between SERS intensity at 592 cm^−1^ and the logarithm of occludin concentration from 0.1 to 1,000 ng/ml. **(D)** Standard curve of the relationship between SERS intensity at 1,332 cm^−1^ and the logarithm of MMP-9 concentration from 0.1 to 1,000 ng/ml.

The SERS sensor analysis of occludin and MMP-9 was conducted via the aforementioned AuMNPs. Figure 1A exhibited magnetic separation for MNPs. The SERS peak at 592 cm^−1^ for NBA-labeled AuMNPs increases with the increasing concentration of occludin from 0.1 to 1,000 ng/ml (Figure 2B). Figure 2C shows that the standard curve between peak intensity at 592 cm^−1^ and the logarithm of occludin concentration was established with a good correlation coefficient of 0.98. Similarly, a sharp increase in the SERS signal at 1,332 cm^−1^ was observed as the MMP-9 concentration increased from 0.1 to 1,000 ng/ml (Figure 2B). Figure 2D illustrates a good linear relationship between intensity at 1,332 cm^−1^ and the logarithm of MMP-9 concentration with a good correlation coefficient of 0.97.

### SERS sensor assay for MMP-9 and occludin concentrations in a mixed system

**FIGURE 3:**
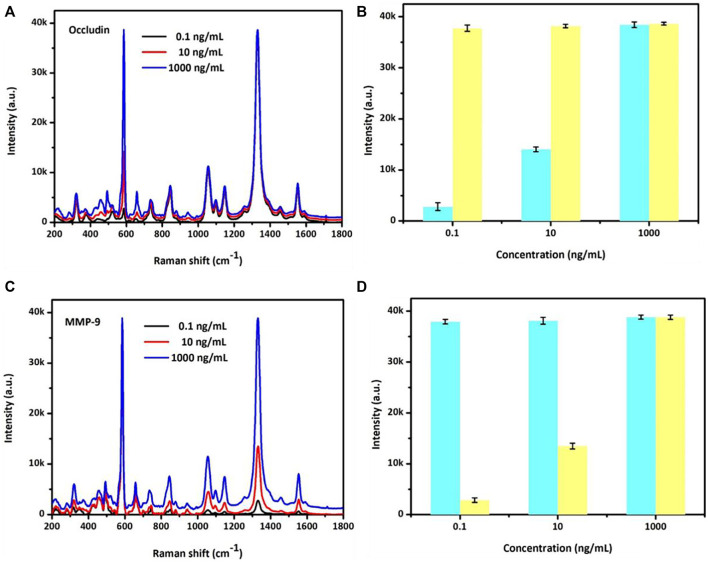
**(A)** Specific analyses of NBA SERS spectra and **(B)** SERS intensities at 592 cm^−1^ with the occludin concentration ranging from 0.1 to 1,000 ng/ml and MMP-9 at 1,000 ng/ml. **(C)** Specific analyses of the DTNB SERS spectra and **(D)** SERS intensities at 1,332 cm^−1^ with the MMP-9 concentration ranging from 0.1 to 1,000 ng/ml and occludin at 1,000 ng/ml.

The SERS sensor analysis for a mixed system of occludin and MMP-9 was also detected. As shown in Figure 3, adding occludin and MMP-9 into the mixed system, the SERS peak intensity at 592 cm^−1^ of NBA-labeled AuMNPs increased with the concentration increase of occludin, while the intensity of the SERS peak at 1,332 cm^−1^ of DTNB-labeled AuMNPs showed no obvious changes for constant 1,000 ng/ml of MMP-9. Similarly, in the mixed system, an increase of SERS peak intensity at 1,332 cm^−1^ for DTNB-labeled AuMNPs was observed with the increasing concentration of MMP-9, and the peak intensity at 592 cm^−1^ for NBA-labeled AuMNPs remained unchanged for constant 1,000 ng/ml of occludin. The specific relationship between SERS intensity and concentration of occludin as well as MMP-9 is presented in Figures 3B–D. In addition, compared with the single system, the position of the SERS peak was still fixed at 592 and 1,332 cm^−1^. All the aforementioned results indicated that there is no cross-reaction between occludin and MMP-9 in the mixed system.

### Evaluation of neurological function and SERS intensity of occludin and MMP-9 in a rat SAH model

**FIGURE 4:**
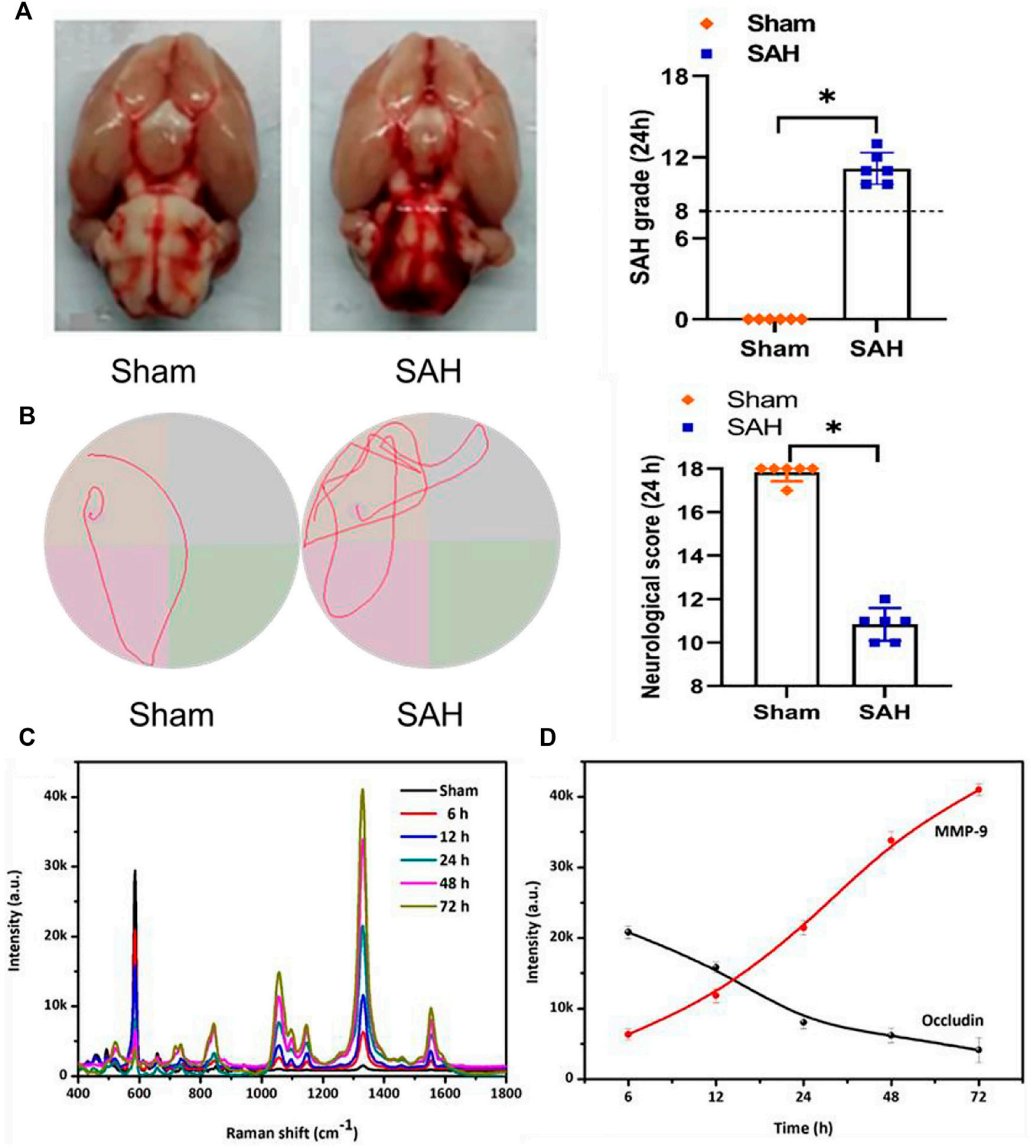
**(A)** Photograph of rat brains and SAH grading scores in the sham and SAH groups. The photograph of MWM trails in the sham and SAH groups. **(B)** Specific neurological score at 24 h in the sham and SAH groups. **(C)** SERS spectra and **(D)** SERS intensity curve of NBA corresponding to the time-dependent concentrations of occludin and DTNB corresponding to the time-dependent concentrations of MMP-9 in the blood samples of the rat SAH model.

No statistical differences in body temperature, arterial blood pressure, body weight, and arterial blood gas were found in the different experimental groups. Figure 4A shows the general picture of the brain in the sham and SAH groups, which exhibits blood clots on the surface of the brainstem and the area of the circle of Willis in the SAH group, and specific SAH grades manifested that the grade score of the SAH group statistically reduced compared with that of the sham group. The mortalities of rats in the sham group was 0% (0/20 rats), and in the SAH group it was 30% (6/20 rats). We also conducted Morris water maze trials to test the difference of spatial learning deficits, and the result demonstrated that, compared with the sham group, the swimming distance and escape latency were significantly increased in the SAH group (Figure 4B). Furthermore, the details of the modified Garcia scoring system are presented in Supplementary Table S1, and the result indicates that the neurological score of the SAH group statistically reduced when comparing with that of the sham group (Figure 4B).

After confirming the selectivity and sensitivity of the SERS sensor strategy in *in vitro* research, we further examined the levels of occludin and MMP-9 in the blood samples of rat SAH models using SERS sensors. As presented in Figures 4C,D, the intensities of the characteristic peak showed a time-dependent decrease at 592 cm^−1^ and a time-dependent increase at 1,332 cm^−1^ after adding the blood samples, indicating that the content of occludin gradually decreased and that of MMP-9 gradually increased over time, which was consistent with the findings of the previous study (Altay et al., 2012; Egashira et al., 2015; Zhou et al., 2015).

### SERS sensor detection of occludin and MMP-9 in SAH patients

**FIGURE 5:**
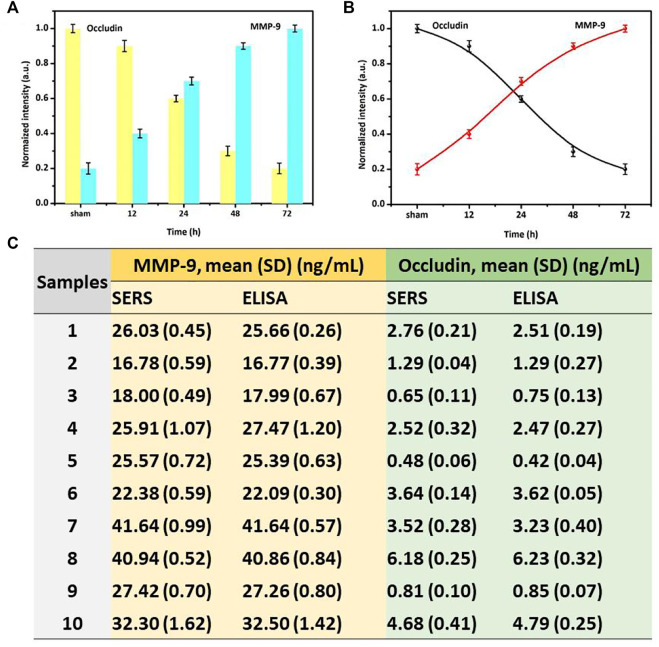
**(A)** Specific-normalized SERS intensities and **(B)** SERS-normalized intensity curve of NBA corresponding to the time-dependent concentrations of occludin and DTNB corresponding to the time-dependent concentrations of MMP-9 in blood samples of SAH patients. **(C)** Specific concentration results of MMP-9 and occludin obtained by ELISA and SERS assays in different samples.

The SERS sensor strategy was also used to quantify occludin and MMP-9 in the blood samples of SAH patients. Figure 5 suggested the specific normalized intensity and the intensity trend of occludin and MMP-9 are in accordance with those in rat SAH models. The results showed that the normalized SERS intensity of occludin was gradually decreased and the corresponding intensity of MMP-9 increased over time. In this study of clinical samples, we adopted the normalized intensity to facilitate the comparison between groups. To verify the accuracy of this method, ELISA and SERS were stochastic and simultaneously used to quantify the concentration of MMP-9 and occludin in the blood samples. We further calculated the average levels of MMP-9 and occludin in 10 samples. All calculated concentrations of MMP-9 and occludin in Figure 5C indicated that the results acquired from SERS detection were in agreement with those obtained using ELISA. However, the procedure for SERS analysis is simpler in antibody modification and test reaction than in the ELISA assay, and the antibody requirement for Raman detection is insignificant for its high sensitivity. Thus, the SERS-embedded AuMNPs would help in potentially identifying low-abundance MMP-9 and occludin with high accuracy and tracking the dynamic changes of these molecules for early diagnosis and prognosis assessment.

## Conclusion

In summary, we established sensitive and accurate SERS tags based on SERS label-embedded AuMNPs for the first dynamic monitoring quantitative double detection and an SERS sensor of MMP-9 and occludin in SAH patients. GNSs with well-defined dendrites and uniform sizes were synthesized and contributed to the enhancement effect of the SERS tags by the nanogaps between them. When combining the NBA-labeled AuMNPs with occludin or DTNB-labeled AuMNPs with MMP-9, a linear SERS signal relationship can be achieved with the detection range from 0.1 to 1,000 ng/ml and the stable limit of detection (LOD) of 0.1 ng/ml. For ELISA, the detection range of MMP-9 and occludin was 1.5–48 and 0.1–20 ng/ml, respectively. Two kinds of SERS labels were used for the sensitive and reliable detection of MMP-9 and occludin, without a time-consuming reaction and complex operation when comparing with conventional ELISA. The SERS results revealed that a decreased concentration of occludin and an increased concentration of MMP-9 can be found after SAH, which was consistent with the results obtained by ELISA. The method proposed in this study could offer a powerful strategy for multiple screening and quantification of MMP-9 and occludin in *in vitro* and blood samples, which has great potential in clinical practice for SAH severity evaluation and prognosis assessment.

## Data Availability

The raw data supporting the conclusions of this article will be made available by the authors, without undue reservation.
