# Supplementary material for: Rapid magnetic separation: An immunoassay platform for the SERS-based detection of subarachnoid hemorrhage biomarkers
Source: Front Chem. 2022 Oct 21;10:1002351. doi: 10.3389/fchem.2022.1002351 (PMC9634124; doi:10.3389/fchem.2022.1002351)
Supplement: Supplementary file 1 [file DataSheet1.docx]

**Supplementary Materials**

**Rapid magnetic separation: An immunoassay platform for the SERS-based detection of subarachnoid hemorrhage biomarkers**

*Ying Wang^1^, Jingyi Sun^2^, Peng Zhao^1^, Hui Yi^1^, Hui Yuan^3^, Mingfeng Yang^3^, Baoliang Sun^1*^, Fengyuan Che^1*^*

*^1^ Linyi People’s Hospital; Shandong First Medical University & Shandong Academy of Medical Sciences, Taian, Shandong, 271000, China*

*^2^ Shandong Provincial Hospital Affiliated to Shandong First Medical University & Shandong Academy of Medical Sciences, Jinan, Shandong, 250021, China*

*^3^ Second Afﬁliated Hospital; Shandong First Medical University & Shandong Academy of Medical Sciences, Taian, Shandong, 271000, China*

**
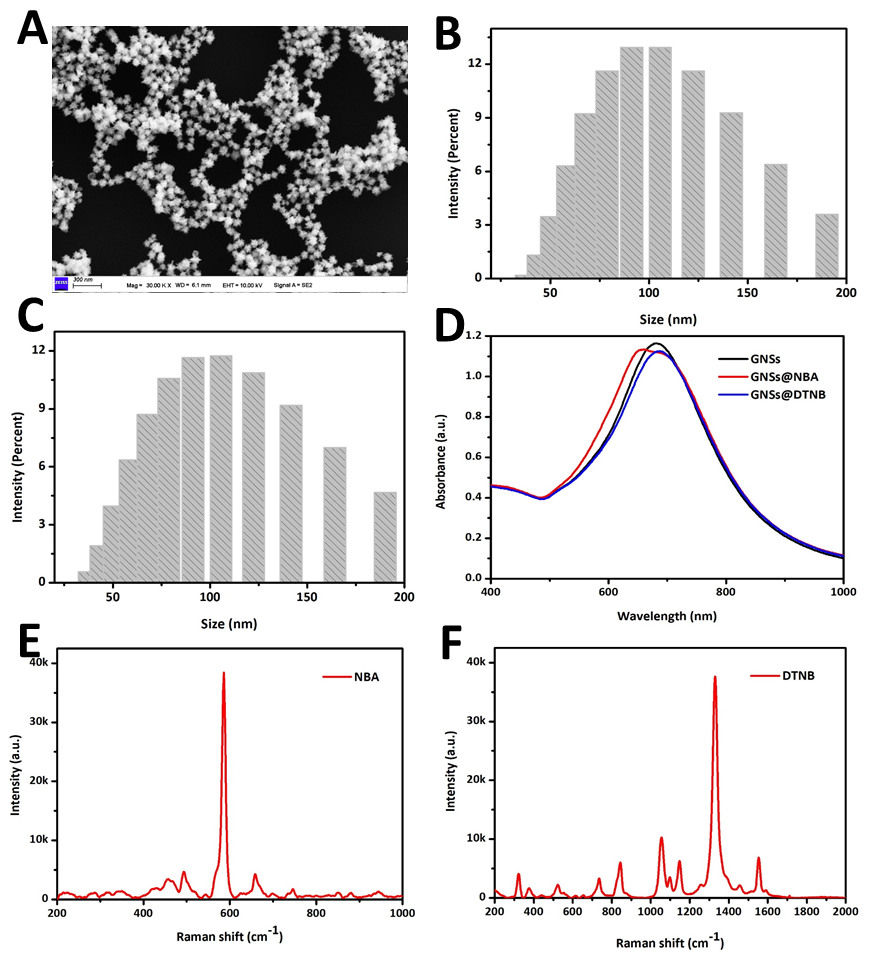
**

***Figure S1.*** *(A) SEM images of the GNSs. (B) DLS measurement for GNSs. (C) DLS measurement for GNSs after modifying signal molecule. (D) UV-Vis-NIR spectra of the GNSs. (E) SERS spectra of NBA labeled GNSs. (F) SERS spectra of DTNB labeled GNSs.*

In this task, the synthesis gold nanofloers the surface enhancement factor (EF) is 5×10^6^. It is calculated by using the analytical chemistry point of view through the analytical EF (AEF) defined as

*AEF = (I_SERS_／C_SERS_)／(I_RS_／C_RS_)*

Where ISERS corresponds to the Raman intensity obtained for the GNS under a certain concentration CSERS and IRS corresponds to the Raman intensity obtained under non-SERS conditions at an analyte concentration of CRS. The experimental conditions, such as the laser wavelength, laser power, microscope objective or lenses, spectrometer, and measuring conditions on the substrate, are taken into account and are identical in all cases.


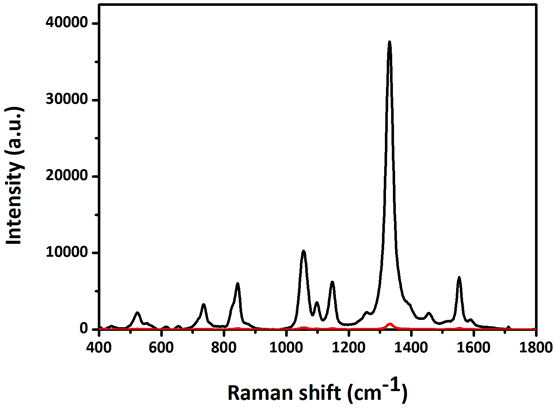


***Figure. S2.*** *The detailed calculation about SERS enhancement factor of nanoparobes.*

| **Table S1** Modified Garcia scoring system evaluating neurological score in rat SAH model | | |
| --- | --- | --- |
| Tests | Standards of grading | |
| Voluntary movement (Put the rat in the cage and observe for 5 minutes) | 0: No voluntary movement; 1: Rarely moves; 2: Moves but only touches at most one cage wall; 3: Moves and touches at least three cage walls | |
| Symmetry of posture (Hang the rat in the air and observe the state of the limbs) | 0: No movement on the affected side; 1: Slight movement on the affected side; 2: Movement of the affected side is slow; 3: The symmetry of bilateral posture is good | |
| Forelimb Stretching movement (Hang the tail to make the hind limbs hang in the air, make them walk on the table by forelimbs only, and observe the forelimb extension) | 0: No extension of the affected forelimb; 1: Slight extension of the affected forelimb; 2: Stretching exercises for the affected forelimb but less than the healthy side; 3: The symmetrical extension of both sides is good |  |
| Climbing wall of cage | 1: Fails to climb; 2: Climbing weakly on the affected side; 3: Normal climb | |
| Tactile reflex on both sides of the body | 1: No response on affected side; 2: Weaker response on affected side than the healthy side; 3: Same response on both sides | |
| Tactile reflection of the beard on both sides | 1: No response on affected side; 2: Weaker response on affected side than the healthy side; 3: Same response on both sides | |
